# Effects of yoga, strength training and advice on back pain: a randomized controlled trial

**DOI:** 10.1186/s12891-017-1497-1

**Published:** 2017-03-29

**Authors:** Elisabeth Björk Brämberg, Gunnar Bergström, Irene Jensen, Jan Hagberg, Lydia Kwak

**Affiliations:** 1 Unit of Intervention and Implementation Research for Worker Health, Institute of Environmental Medicine, Nobels väg 13, Karolinska Institutet, 171 77 Stockholm, Sweden; 2 Centre for Occupational and Environmental Medicine, Stockholm County Council, Solnavägen 4, 113 65 Stockholm, Sweden

**Keywords:** Randomized controlled trial, Back pain, Yoga, Strength training, Sickness absenteeism, Sickness presenteeism, Disability

## Abstract

**Background:**

Among the working population, non-specific low-back pain and neck pain are one of the most common reasons for sickness absenteeism. The aim was to evaluate the effects of an early intervention of yoga - compared with strength training or evidence-based advice - on sickness absenteeism, sickness presenteeism, back and neck pain and disability among a working population.

**Methods:**

A randomized controlled trial was conducted on 159 participants with predominantly (90%) chronic back and neck pain. After screening, the participants were randomized to kundalini yoga, strength training or evidence-based advice. Primary outcome was sickness absenteeism. Secondary outcomes were sickness presenteeism, back and neck pain and disability. Self-reported questionnaires and SMS text messages were completed at baseline, 6 weeks, 6 and 12 months.

**Results:**

The results did not indicate that kundalini yoga and strength training had any statistically significant effects on the primary outcome compared with evidence-based advice. An interaction effect was found between adherence to recommendations and sickness absenteeism, indicating larger significant effects among the adherers to kundalini yoga versus evidence-based advice: RR = 0.47 (CI 0.30; 0.74, *p* = 0.001), strength training versus evidence-based advice: RR = 0.60 (CI 0.38; 0.96, *p* = 0.032). Some significant differences were also found for the secondary outcomes to the advantage of kundalini yoga and strength training.

**Conclusions:**

Guided exercise in the forms of kundalini yoga or strength training does not reduce sickness absenteeism more than evidence-based advice alone. However, secondary analyses reveal that among those who pursue kundalini yoga or strength training at least two times a week, a significantly reduction in sickness absenteeism was found. Methods to increase adherence to treatment recommendations should be further developed and applied in exercise interventions.

**Trial Registration:**

Clinicaltrials.gov NCT01653782, date of registration: June, 28, 2012, retrospectively registered.

## Background

The general lifetime prevalence of back pain is estimated to be 70–80% and of neck pain 30–40% [1, 2]. Among the working population, non-specific low-back pain (LBP) and neck pain are one of the most common reasons for sickness absenteeism (SA) [3] and sickness presenteeism (SP), i.e., going to work despite illness [4]. They are associated with considerable costs for the individual, employers and society [5].

An intervention that has demonstrated promising effects on reducing low back pain [6, 7], improving back disability [8, 9] and reducing neck pain [10] is yoga. Yoga is also cost-effective in improving health related quality of life for patients suffering from such pain [11, 12]. Less is known about the effect of yoga on SA or return to work. One study found no significant differences in SA when yoga was compared with a control group which received written information about a healthy lifestyle [13]. We are not aware of any previous studies into the effects of yoga on SP. However, interventions that include physical and psychological activity (i.e. applied relaxation) have been shown to have a positive impact on SA [14]. As yoga contains both these components, its potential beneficial effect on SA and SP should be tested.

The shortcomings of previous studies of yoga include the lack of adequate reporting of intervention components [15] and the lack of active comparison groups [16]. A suitable active comparison group could be strength training, which is used with positive effects on CLBP [17] and neck pain [18]. As strength training, unlike yoga, only involves physical activity, the addition of psychological activities, as in yoga, could prove more effective and needs to be explored. The positive effect of yoga on the emotions, sleep and cognitive processes [19], and its effectiveness in reducing pain and depression in somatization disorders including chronic pain conditions [20], further underscores this assumption.

In the present study we want to further the understanding of this area by comparing two active interventions, namely yoga and strength training, with a minimal intervention containing evidence-based advice. The interventions can be regarded as early interventions in relation to the duration of sick-leave for back and neck pain because they are administered early in the sick-leave process (<8 weeks of sick-listing). Previous sick leave is a strong risk factor for long-term sick leave and a risk for disability pension [21, 22]. The cost-effectiveness of the intervention has been reported in Aboagye E, Karlsson ML, Hagberg J and Jensen I [12].

### Aim and hypothesis

The aim was to evaluate the effects of an early intervention of yoga - compared with strength training or evidence-based advice - on sickness absenteeism, sickness presenteeism, back and neck pain and disability among a working population suffering from low back pain with or without neck pain.

#### H1

Kundalini yoga and strength training are superior to evidence-based advice alone in reducing the primary outcome of sickness absenteeism at 12 months, and the secondary outcomes of sickness presenteeism at 12 months, LBP and neck pain intensity and disability at 6 months.

#### H2

Kundalini yoga is superior to strength training in reducing the primary outcome of SA at 12 month follow-up.

#### H3

Kundalini yoga is superior to strength training in reducing the secondary outcomes of SP at 12 month follow-up, LBP and neck pain intensity and disability at 6 month follow-up.

## Methods

### Design and overview

The study was a randomized controlled trial employing a 3 × 4 repeated-measures design with three groups and four assessment periods (pre-intervention, 6 weeks, 6-month and 12-month follow-up). This study focuses on baseline, 6-month and 12-month follow-up. The three groups were kundalini yoga, strength training and a minimal-intervention (advice only) control group (CG). The study is registered in the Clinical Trials protocol registration system (NCT01653782). The authors confirm that all ongoing and related trials for this intervention are registered.

The study used a block randomization design. A random allocation sequence was generated by the statistician (JH). For each participant an opaque envelope was opened, in consecutive order, by an external research assistant not involved in the inclusion process. The participants did not know the content of the different intervention arms. The yoga leader and physiotherapist were not blinded. The research group assessing the study’s outcome was blinded during the data collection and data analysis.

After randomization the participants received information about the offered intervention from a research assistant not involved in the offered interventions. Yoga, strength training and evidence-based advice were all presented as well-established interventions, in order to equalize the participants’ expectations.

### Power

When planning the study, the goal was to detect a 25% reduction in the primary endpoint SA with 80% power. Sample size was determined by a priori power-calculation based on the number of participants and effects from previous studies [23, 24] with similar study objects and the same outcome measures. The calculated group size was 40 participants per group to estimate an effect of 25% regarding changes in SA.

### Participants

Participant recruitment and follow-up were performed from April 2010 to June 2012. During the period April to September 2010, participants (*n* = 8) were recruited from OHS-units in Stockholm County, Sweden, in accordance with the original plan. However, due to the low influx of persons with back and neck pain from the OHS, participants were thereafter recruited by advertising in local media (*n* = 302).

The inclusion criteria were the presence of non-disabling, non-specific LBP, with or without neck pain, and a score of ≥ 90 points on the Örebro Musculoskeletal Pain Screening Questionnaire (OMPSQ) [25]. Participants were furthermore to be 18-60 years of age and proficient in Swedish. Non-disabling was defined from the perspective of work disability. To be included in the study individuals were not to be on sick leave or, if they were on sick leave, for less than 8 weeks. Based on findings by Linton SJ and Boersma K [26] a cut-off score of ≥ 90 points was chosen, as this cut-off point has the highest sensitivity in predicting long-term sick leave (89%) due to back pain. Since the Swedish sick leave system grants sickness benefit also to students and unemployed, they were also eligible for participation in the study. The exclusion criteria were spinal pathology (e.g. tumours or spinal fractures), continuous ongoing sick-listing ≥ 8 weeks, comorbidities that could affect the ability to fully participate in the study (e.g., physical disability, psychosis), existing weekly yoga practice or strength training and verified pregnancy.

The screening procedure was divided into four steps:

1. The OMPSQ [25] was mailed to those who responded to the invitation to participate
2. Individuals meeting the inclusion criteria and who scored ≥ 90 on the OMPSQ were invited to undergo a physical examination
3. The examination was performed by an orthopaedic specialist or a licensed chiropractor, and included screening for red flags and spinal pathology
4. Individuals meeting the exclusion criteria after the physical examination were excluded from the study. The remaining individuals were randomized to one of the three groups.

### Interventions and control condition

A minimal intervention comprising self-care advice was given to all participants.

### Control group (CG)

The participants received a booklet - ‘The Back Book’ - containing evidence-based advice that encourages strategies for self-care, information on medication, sick leave and strategies for managing pain. Participants were also given a verbal, evidence-based recommendation to stay active by an orthopaedic specialist. The participants did not receive any further attention and they did not gather as a group. This form of brief counselling has been shown to have positive effects on sick leave among individuals with CLBP [27, 28] and is “best practice” for non-specific back pain [29].

### Kundalini yoga

In Kundalini yoga, the starting position can be sitting, lying, or standing, and the movements are generally slower than in traditional forms of yoga. As kundalini yoga not only involves a physical activity component but also a psychological component, i.e. meditation and awareness training (focusing on thoughts, breathing, postures etc), which is also likely to affect perceived pain and disability [30], we expect yoga to have a greater impact on LBP, neck pain, SA and SP than strength training.

A prestructured standardized kundalini yoga programme adapted for back pain was used. The intervention lasted for six weeks with yoga classes twice a week. The classes were held at a yoga studio, performed in groups and led by an experienced yoga instructor. The dosage of instructor-led yoga was about 60 min/class twice weekly.

Two yoga programmes were used during the intervention: one with five yoga postures (breath of fire, spine flex, spine twist, spine side bend and neck roll) and one with nine yoga postures (spine flex in easy pose, spine flex in rock pose, spine twist, bear grip, spine twist with locked elbows, shoulder lifts, neck roll, alternate bear grip and sat kriya). Every posture was performed for between 1 – 3 min with 1 – 2 min of rest before a new posture was performed. The instructor alternated between these two programmes and accordingly each programme was performed weekly in the classes. Two sorts of breathing technique were used in all classes: long deep breathing and breath of fire. The programme also included a few minutes of applied relaxation after the yoga practice.

In addition to the classes, participants were encouraged to do home practice by performing the yoga programmes as often as possible, at least twice a week. The participants received a CD with instructions and written information, including drawings of each posture. At the end of the intervention, i.e. after six weeks, the participants were encouraged to continue practicing the yoga programmes at least twice a week.

### Strength training

As suggested by Park et al. [16] we used an active comparison group. The intervention period was six weeks with five supervised strength-training sessions, led by an experienced physiotherapist. The participants received an individually tailored, physiotherapist-supervised strength training programme with home practice. The participants received a total of five sessions of 60 min. The participants were told to perform the strength training programme at least two times per week. The home practice was guided by written material and a follow-up phone call from the physiotherapist eight weeks after the intervention period.

The programme focused on muscle strengthening, endurance, stabilization for the core muscles and body awareness. Each exercise was repeated 18 – 24 times with 30 s of rest in-between, in two sets. Machine and hand weights and an exercise ball were used. At sessions one and two (intervention week 1) the programme was individually adapted; resistance and intensity were modified according to each participant’s capacity. The intensity and resistance were gradually increased during the intervention, starting with low intensity/resistance at the first session and thereafter increasing at the following sessions (intervention week 2, 4 and 6).

### Primary and secondary outcome measures

Data was collected using Web-based, validated questionnaires and SMS -text messages [31]. Persons involved in the interventions were not involved in the data collection.

### Sickness absence (SA)

Information on SA was measured using the following SMS-administered question ‘How many days in the past four weeks have you been absent from work because of illness? Answer with a number between 0 and 31’. The SMS-messages were administered by SMS-track (https://sms-track.com/) monthly for 12 months, except for the intervention period, when the data were completed weekly for six weeks. Self-reported SA was chosen because the Swedish Social Insurance Board (SSIB) only covers SA of longer than 14 days’ duration; SA ≤ 14 days is covered by the employer and is thus not recorded in the SSIB register. Self-reported SA has been shown to have acceptable reliability [32].

### Sickness presenteeism (SP)

SP was measured at baseline and at the 12-month follow-up, by means of the following question: ‘Did you, during the last 6 months go to work despite feeling that you really should have taken sick leave because of your state of health?’ [33]. The response format was 1) No, never, 2) Yes, once, 3) Yes, 2 to 5 times, 4) Yes, > 5 times. This item has been extensively used in previous research [4, 33].

### Back and neck pain

Back and neck pain intensity and disability were measured at baseline and at the 6-month follow-up, by the Chronic Pain Grade Scale (CPGS), a validated and established 7-item instrument [34]. In the Swedish version, the CPGS is divided into back and neck pain intensity and disability. The response format is an 11-point Likert scale ranging from 0-10. Following the scoring protocol from von Korff [34], two subscales were calculated: pain and disability. The subscales are calculated as the mean intensity and transformed into 0-100. The CPGS questionnaire was administered at baseline and at 6-month follow-up. The 12-month follow-up included items related to the study’s outcomes SA and SP. The reasons for this were to minimize the questionnaire burden on the respondents.

### Adherence to treatment recommendations

The participants’ adherence to the recommendations was self-reported by SMS-text messages once a week for six weeks during the intervention period. The participants in the active interventions responded to questions about the intervention they had been given during the intervention period. The participants who received evidence-based advice were instructed to respond in relation to overall exercise. The text message question was: ‘How many times have you exercised in the past week? Answer with a number between 0 and 7’. Thereafter, the text message was sent once a month with the question: ‘How many times have you exercised in the past four weeks? Answer with a number between 0 and 31’. This was repeated until the end of the study (after 12 months).

### Statistical analyses

The statistical analyses were carried out using SPSS 22.0. An intention-to-treat analysis was conducted which included all randomized individuals, irrespective of whether they had adhered to the intervention programme or not. Two conservative imputation analyses were performed on the primary outcome sickness absenteeism. In the first analysis, we used the relative frequencies of zeros and ones in each group to randomly generate zeroes and ones for the drop-outs. In the second analysis we used “Last value carried forward”. Both analyses generated similar results (data not presented) to those presented in the results section.

### Primary analyses

The primary outcome SA was dichotomized as follows: data on SA were transformed to three four month-time periods: time period 1 (months 1-4), time period 2 (months 5-8) and time period 3 (months 9-12). Data should have been reported for at least 2 months per time period. Due to the skewed distribution, the SA was dichotomized. For each time period the SA was coded as: 0 (0) day, ≥1 (1) day/days. These cut-points are based on the fact that it is common to detect a U-shaped distribution with high frequencies of SA close to zero, high frequencies of SA close to SA maximum and low frequencies in between.

Relative risks (RR) of SA were calculated using modified Poisson regression in generalized estimating equation (GEE). The GEE generates one RR even if the outcome has more than one sample period. RR was calculated for SP using modified Poisson regression. The CG was the reference group against the intervention groups. When comparing the active interventions, yoga served as the reference group.

For the secondary outcomes, the SP variable was dichotomized as follows: 0 to 1 time (0), ≥ 2 times (1), since previous research has revealed that a cut-off at 2 times is associated with further health consequences [35, 36]. Regarding back and neck pain intensity and disability, a linear model was applied to analyze between-group comparisons of differences in von Korff’s scores at the 6-month follow-up, adjusted for baseline values on the respective outcome variables.

### Secondary analyses

Initial analyses revealed a significant interaction effect between the primary outcome SA, number of times exercised per week and intervention group (yoga *p* = 0.015; strength training *p* = 0.018). The Relative Risk (RR) for yoga versus the control group was significantly < 1 for exercising more than 2 times/week (*p* = 0.0496 at exercise = 2.1 times/week). The RR for strength training versus control was significantly < 1 for training 3 times/week (*p* = 0.0492 at exercise = 3 times/week). Thus, the main analyses were re-analyzed introducing the interaction terms.

Based on the lowest detected interaction point, number of times exercised was dichotomized into < 2 times/week (non-adherers) and ≥ 2 times/week (adherers). The dichotomization is also supported by the global physical activity recommendations of muscle-strengthening activities 2 or more days a week. All analyses were performed separately for non-adherers and adherers.

## Results

**Fig. 1:**
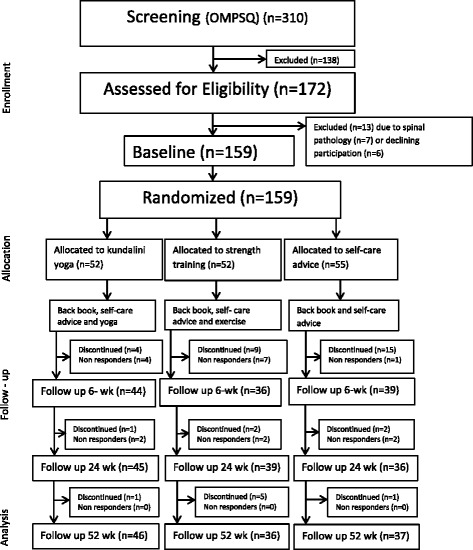
Flow chart

A total of 310 subjects were screened for eligibility. Of those, 172 met the inclusion criteria and were offered the physical examination. Of these, 13 were excluded (spinal pathology *n* = 7) or due to declined to participate further (*n* = 6). Consequently, 159 individuals gave their written consent to participate and were randomized to the three groups: yoga, *n* = 52; strength training, *n* = 52; CG, *n* = 55. A flow-chart of the study is presented in Fig. 1.

Of the 159 participants who were allocated to the three groups, 119 (74.8%) responded to the questionnaires on all three occasions (yoga, 46; strength training, 36; CG, 37) (see Fig. 1). Seventy-one percent of participants were women; the mean age at baseline was 45.7 years (SD 10.3); the mean neck pain intensity was 41.9 (SD 25.2) and the mean back pain intensity was 55.0 (SD 18.2). In Fig. 1, the group of those who discontinued refers to the participants who chose to discontinue their participation in the study. Non-responder refers to those who did not respond to the follow-up but continued their participation in the study.

Descriptive information for the subjects participating in each group and for the CG is presented in Table 1. There were no significant differences between the intervention groups regarding the baseline values age, sex, education, years lived in Sweden, sickness presenteeism, back and neck pain intensity and disability. As shown in Table 1, the majority (more than 90%) of the sample had CLBP. Hereafter the sample will be collectively referred to as suffering from CLBP, based on the definition of Von Korff M and Saunders K [37].

**Table 1:** Participants’ baseline characteristics

Description	Kundalini yoga (*n* = 52)	Strength training (*n* = 52)	Control group (*n* = 55)
Female n, (%)	37 (71.7)	32 (61.5)	44 (80)
Age mean, (SD)	46.9 (9.6)	46.3 (9.3)	43.9 (11.7)
Marital status n, (%)^a^			
Single	18 (34.6)	17 (33.3)	11 (20.8)
Cohabitant with partner/married	31 (59.6)	29 (56.9)	34 (64.2)
Cohabitant with others	2 (3.8)	4 (7.8)	7 (13.2)
Education n, (%)^b^			
Compulsory school	3 (5.8)	3 (5.8)	4 (7.5)
Upper secondary school	22 (42.3)	26 (50.0)	28 (52.8)
University/University college	27 (51.9)	23 (44.2)	21 (39.7)
Lived in Sweden n, (%)^c^			
Always	37 (71.2)	38 (73.1)	35 (66.0)
Less than 5 years	2 (3.8)	1 (1.9)	1 (1.9)
More than 5 years	13 (25.0)	13 (25.0)	17 (32.1)
BMI mean, (SD)	25.7 (4.3)	26.0 (4.2)	25.2 (4.2)
Employment status *n*, (%)^d^			
Employed	41 (78.8)	45 (86.5)	53 (96.4)
Student/unemployed/unpaid work	10 (19.2)	7 (13.4)	2 (3.6)
Chronic low back pain *n*, (%)^e^	48 (94)	50 (96)	51 (93)

^a^Marital status missing in kundalini yoga: 1, strength training: 2, control group: 3. ^b^Education missing in control group: 2. ^c^Lived in Sweden missing in control group: 2. ^d^Employment status missing in kundalini yoga: 1. ^e^The definition of acute, sub-acute and chronic pain is based on the duration of pain. Chronic low back pain is defined as having pain more than 12 weeks (Von Korff & Saunders, 1996). *Abbreviation SD* standard deviation

### Attrition rates

Forty participants were lost to follow-up, i.e. those who discontinued and the non-responders (yoga 6 (11.5%); strength training 16 (30.7%); CG 18 (32.7%)) at the 12-month follow-up. The yoga group had a statistically significantly lower loss to follow-up than the strength training and CG (Fisher’s exact test = 0.017). Sixty percent of those lost to follow-up were women, mean age was 42.0 years (SD 11.3), mean neck pain intensity was 45.0 (SD 25.3) and mean back pain intensity was 61.6 (SD 14.8). However, there were no significant differences between those who were lost to follow-up and participants in terms of age, sex, or pre-intervention values on neck and back pain. For the SMS assessments for SA, the response rate for T1 was 82.4%, T2 79.2% and T3 74.4%. At the 6 month follow-up the response rate for the CPGS questionnaire was 78.0%.

### Adherers/non-adherers to recommendations

The proportion of participants who adhered to the recommendations (exercised at least 2 times/week) during the 6 month follow-up was: 54% (yoga), 34% (strength training) and 42% (CG).

### SA and SP at the 12-month follow-up

Table 2 shows descriptive information and the results of the modified Poisson regression analysis for the outcomes SA and SP for comparisons between the intervention groups versus the CG. The results are presented as RRs with 95% confidence intervals in parenthesis.

**Table 2:** Descriptive information and risk ratio estimates on sickness absenteeism and sickness presenteeism

	Kundalini yoga (*n* = 46)	Strength training (*n* = 36)	Control group (*n* = 37)
Sickness absenteeism			
*Time period 1*			
Mean in days/median/SD	4.1/1.4/7.7	5.0/0.4/9.1	8.9/1.7/20.7
0 time (%)	43.5	50.0	34.1
≥ 1 time (%)	56.5	50.0	65.9
*Time period 2*			
Mean in days/median/SD	4.0/0.0/8.4	6.4/2.6/14.7	12.0/2.6/25.0
0 time (%)	54.5	25.6	28.2
≥ 1 time (%)	45.5	74.4	71.8
*Time period 3*			
Mean in days/median/SD	3.6/0.1/6.3	9.5/0.1/22.2	9.2/0.0/23.1
0 time (%)	50.0	48.7	55.6
≥ 1 time (%)	50.0	51.3	44.4
Risk ratio estimate (CI)	0.82 (0.63; 1.08)	0.95 (0.73; 1.22)	
Sickness presenteeism^a^			
*Baseline, n* (%)			
0 - 1 times	17 (34.7)	14 (26.9)	25 (49.0)
≥ 2 times	32 (65.3)	38 (73.1)	26 (51.0)
*Follow-up 12 months,* n (%)			
0 - 1 times	24 (53.3)	13 (36.1)	18 (48.6)
≥ 2 times	21 (46.7)	23 (63.9)	19 (51.4)
Risk ratio estimate (CI)	0.77 (0.49; 1.21)	1.06 (0.71; 1.57)	

Descriptive information and risk ratio estimate with 95% confidence intervals on the outcomes sickness absenteeism and sickness presenteeism for comparisons between the kundalini yoga and strength training, versus the control group. ^a^Number of times during the last 6-month period. *Abbreviations SD* standard deviation, *CI* confidence interval

A RR above 1 indicates a higher risk, and a RR below 1 indicates a lower risk of both SA and SP. No statistically significant differences between the intervention arms were found for either SA or SP. A non-significant lower risk of SA was revealed for yoga compared to strength training (RR = 0.86, 0.65; 1.14 (results not presented in Table 2). The corresponding results for SP were: yoga versus CG: RR = 0.77 (0.49; 1.21); strength training versus CG: RR = 1.06 (0.71; 1.58). A non-significant higher risk of SP was revealed for yoga versus strength training (RR = 1.36, 0.92; 2.00) (results not presented in Table 2).

### Back and neck pain intensity and disability at the 6-month follow-up

Descriptive information and results of the ANCOVA on the outcomes back and neck pain intensity and disability for the intervention groups versus the CG, are shown in Table 3.

**Table 3:** Secondary outcomes and parameter estimates for interventions groups versus control group, and between group comparisons

	Kundalini yoga (*n* = 45)	Strength training (*n* = 39)	Control group (*n* = 36)
Neck pain intensity, mean (SD)^a^			
Baseline	44.4 (24.5)	46.5 (24.4)	37.6 (26.1)
6-month follow-up	35.0 (21.1)	29.8 (20.7)	34.3 (27.2)
b (CI)^b^	- 4.8 (- 12.2; 2.5)	- 7.0 (- 14.7; 0.7)	
b (CI)^c^		- 2.35 (- 9.42; 4.72)	
Back pain intensity, mean (SD)^a^			
Baseline	57.1 (18.5)	57.7 (15.4)	55.6 (18.7)
6-month follow-up	47.0 (24.3)	41.7 (20.6)	50.2 (23.9)
b (CI)^b^	- 6.5 (- 14.9; 1.8)	- 9.4 (- 18.1; - 0.8)*	
b (CI)^c^		- 2.89 (- 10.92; 5.14)	
Neck disability, mean (SD)^a^			
Baseline	25.0 (23.3)	28.5 (24.1)	23.7 (22.7)
6-month follow-up	16.3 (20.1)	13.3 (18.3)	21.5 (26.4)
b (CI)^b^	- 8.7 (- 16.0; - 1.3)*	- 9.6 (- 17.2; - 2.0)*	
b (CI)^c^		- 1.01 (- 7.31; 5.30)	
Back disability, mean (SD)^a^			
Baseline	37.2 (23.4)	37.6 (20.9)	38.6 (21.4)
6-month follow-up	29.4 (24.2)	24.8 (24.2)	32.8 (27.8)
b (CI)^b^	- 6.0 (- 15.6; 3.6)	- 9.5 (- 19.3; 0.4)	
b (CI)^c^		- 3.46 (- 12.23; 5.32)	

*p = < 0.05; **p = <0.01; ***p = <0.001. ^a^From the CPGS, ranged from 0 to 100 where higher scores indicate worse pain intensity and disability due to back and neck pain. ^b^ Parameter estimates (b) with parenthesized confidence intervals (95%) from linear model adjusted for baseline scores on the respective outcome variables, comparing kundalini yoga or strength training versus CG. ^c^Estimates of differences from ANCOVA comparing kundalini yoga versus strength training at the 6-month follow-up, kundalini yoga served as the reference group. *Abbreviations SD* Standard Deviation, *CI* Confidence Interval

As shown in Table 3, a significant difference in reduced neck disability was observed for both yoga and strength training compared with the CG. Moreover, a significant difference in reduced back pain intensity was observed for strength training compared with the CG. No other statistically significant findings were observed.

As described in the statistical analyses section, the initial analyses revealed significant interaction effects (Table 4) between the primary outcome SA, number of times exercised per week and intervention group (yoga *p* = 0.015; strength training *p* = 0.018). A secondary analysis was thus conducted in which the interaction term was introduced. In addition, a separate analysis was performed on adherers and non-adherers.

**Table 4:** Sickness absence risk ratio estimates with interaction adjustments for the mean number of exercise times/week

Mean number of exercise times/week	Sickness absence RR Kundalini yoga (95% CI)	Sickness absence RR Strength training (95% CI)
0.0	1.25 (.82; 1.90)	1.43 (.96; 2.13)
0.5	1.11 (.78; 1.58)	1.25 (.91; 1.72)
1.0	.99 (.73; 1.33)	1.09 (.84; 1.42)
1.5	.88 (.67; 1.15)	.95 (.74; 1.23)
2.0	.78 (.60; 1.02)	.83 (.62; 1.11)
2.5	.70 (.52; .94)	.73 (.50; 1.04)
3.0	.62 (.44; .88)	.63 (.40; .99)
3.5	.55 (.36; .84)	.55 (.32; .96)
4.0	.49 (.30; .81)	.48 (.25; .92)
4.5	.44 (.24; .78)	.42 (.20; .90)
5.0	.39 (.20; .76)	.37 (.15; .87)

Sickness absence risk ratio estimate from GEE analyses, with interaction adjustments for the mean number of exercise times/week for the kundalini yoga and strength training, versus the control group at the 12-month follow-up. *Abbreviations RR* risk ratio estimate, *CI* Confidence Interval

As shown in Table 5, significant differences between the intervention groups and the CG were observed within the group of adherers to the recommendations. For the primary outcome the risk of SA during follow-up was reduced by more than 50% for yoga (parenthesized confidence intervals): RR = 0.47 (0.2; 0.7; *p* = 0.001) and 40% for strength training: RR = 0.60 (0.3; 0.1; *p* = 0.032) compared to the CG. No significant differences in SA were found when yoga was compared to strength training.

**Table 5:** Risk ratio estimates for sickness absence for kundalini yoga and strength training, versus control group

	Kundalini yoga versus control group		Strength training versus control group	
Adherence to treatment/week	<2 times/week	≥2 times/week	<2 times/week	≥2 times/week
Sickness absenteeism	RR 1.12 (0.82; 1.52)	RR 0.47 (0.30; 0.74)***	RR 1.12 (0.82; 1.53)	RR 0.60 (0.38; 0.96)*

Risk ratio estimates for sickness absence with parenthesized confidence intervals (95%) from GEE analyses for the kundalini yoga and strength training, versus the control group stratified on adherence to treatment less than 2 times/week or 2 or more times/week, at the 12-month follow-up. *p = < 0.05; ***p = < 0.001. *Abbreviation RR* risk ratio estimate

## Discussion

The aim of the study was to evaluate the effects of early interventions among a working population suffering from LBP with or without neck pain. The interventions under study were kundalini yoga, strength training and evidence-based advice. Where our first hypothesis is concerned, we did not observe that yoga or strength training reduced SA at 12 months more than evidence-based advice alone. Significant effects were observed for yoga on neck disability and for strength training on back pain intensity and neck disability compared with the CG. When the sample was stratified into adherers and non-adherers, the first hypothesis was confirmed, with a significantly reduced risk of SA for yoga and strength training compared with the CG. A possible explanation of the tendency to improvement among the adherers is that an increased dosage of the intervention might result in it having an increased effect. However, the dose of exercise reported by the CG (advice only) had no effect on the outcome. Thus, it seems that increased adherence to the specific exercise taught, i.e. subjects actually doing what the intervention was targeted at in the active interventions, increased the effect. The dose relationship itself was not observed in the CG.

When the active interventions were compared, no significant differences were observed. This is in line with the findings of Sherman and colleagues, who in their two published RCTs comparing yoga, strength training or stretching and a self-care book [38, 39] found that yoga had a positive effect on back-related function at the 26 week follow-up compared with the self-care book. However, yoga was not found to result in greater effects than the strength training or stretching. An explanation may be that exercise alone is not as effective unless accompanied by guidance and practical training instructions. This explanation is supported in a review summarizing the evidence for effective exercise programmes for back pain, in which supervised group treatment, such as the yoga or strength training, combined with a supervised, individually tailored programme, was shown to encourage adherence better than non-supervised treatment [17]. Liddle and colleagues define high or medium adherence as 75%, and low as 15%. Among our participants, the proportions who exercised at least twice times per week during the 6-month follow-up were 54% (yoga), 34% (strength training) and 42% (CG). The intervention conditions did not result in higher adherence than for the minimal intervention. Another explanation for the tendency to improvement among the adherers is that those who received the active interventions learned more effective ways to exercise by means of partly or fully supervision as suggested by Liddle et al. [17] and Falla et al. [40].

Adherence is an important issue to address in forthcoming research as well as address adherence in the analyses of effect. Accordingly, further studies need to evaluate methods for increasing adherence to exercise. Few studies of yoga have included adherence [41]. Adherence to exercise and preventing relapse during follow-up might be supported by regular phone calls, goal-setting, preventive advice, and a self-help workbook [42, 43] as well as supervised exercise sessions with “refresher sessions”, and audio and/or video-recorded instructions for home-use [44, 45].

The effect of yoga and strength training on SA found in the present study is in line with studies that have been conducted into the effect of exercise therapy on SA. These studies have observed and improvement in SA among persons suffering from sub-acute LBP [46] or non-acute LBP [47], even if exercise therapy can encompass different types of intervention and it is unclear exactly which form of exercise therapy is most effective. To our knowledge, no previous studies have evaluated the effect of yoga or strength training on SP. In systematic reviews, yoga has been reported as having promising effects on LBP and disability [6, 7, 9]. Neck pain has been less studied. In one pilot study, yoga was found to result in pain relief and functional improvements compared with evidence-based advice [10]. The differences between our results and previous studies may be explained by the fact that the majority of previous studies have compared yoga with passive controls. Ward L, Stebbings S, Cherkin D and Baxter GD [7] concluded that the effect of yoga is stronger when compared with passive rather than active interventions [7]. The strength training intervention in the present study included supervised strengthening exercises and was individually tailored. These components were observed to have the best outcomes [46, 47].

### Limitations

There is an ongoing debate on subgroup analyses in RCTs. There is, however, a consensus that subgroup analysis, not planned in advance, should be based on analyses of interaction effects, applying risk estimates and interpreted using confidence intervals, rather than p-values only [48–51]. We have adhered to this in our analyses since the observed interaction effect was not foreseen and subgroups were therefore not pre-specified. The statistical power was low in the subgroup analyses, as indicated by the wide confidence intervals. Nonetheless, in order to confirm (or refute) the results of this study it should be replicated applying pre-planned power calculations based on the subgroups identified.

In the present study, the yoga differed from the strength training in the amount of received time and attention, which has to be taken into consideration. There was also a significantly lower loss to follow-up in the yoga group than in the strength training group and CG. However, when we compared yoga with strength training, there were no significant differences on the primary or secondary outcomes. There was a higher loss to follow-up among those with back pain in the CG than in the other groups. Due to the fact that the majority (more than 90%) of the participants suffered from CLBP it was not possible to conduct the analyses on different durations of LBP. This has implications for future research.

The study used validated questionnaires and measurement methods including Web-based self-report questionnaires and SMS text messages. Information on SA and adherence was gathered using SMS track which has been evaluated in several previous studies [31]. This method facilitates repeated measurements with short recall periods, which is thought to reduce recall bias. However, one of the limitations of the study is the 6-month recall period for reporting SP. The questions about SA and SP were not pain specific because previous studies have shown that subjects suffering from back pain have an increase in sick leave due to other diagnoses varying over time. The questions about adherence did not distinguish between session attendance and home practice during the intervention period. As a consequence we are unable to draw any conclusions about the importance of where the exercise was performed. In the present study data regarding adherence to treatment recommendations was collected in number of days instead of number of minutes. To gain a better understanding of the dose effect relationship, we recommend future research to collect data in number of minutes. The small percentage of men in the study is a limitation for the generalizability of the results.

## Conclusions

This study demonstrates the importance of addressing adherence when studying exercise interventions. Adherence was shown to have a significant interaction effect with type of intervention and outcome. When adherence was not taken into consideration, the overall results did not reveal any significant differences between the groups for the primary outcome SA. Where the secondary outcomes SP, back and neck pain intensity and disability are concerned, the active interventions showed some significant effects compared with the CG. This indicates that it is important to measure adherence to treatment recommendations as well as to distinguish between different forms of adherence (for example session attendance or adherence to recommendations for home practice) and further develop methods which encourage adherence in exercise interventions.

## Abbreviations

ANCOVA: Analysis of covariance
CG: Control group
CI: Confidence interval
CLBP: Chronic low back pain
CPGS: Chronic pain grade scale
GEE: Generalized estimation equation
LBP: Low back pain
RCT: Randomized controlled trial
RR: Risk ratio estimate
SA: Sickness absenteeism
SD: Standard deviation
SP: Sickness presenteeism
SPSS: Statistical package for the social sciences
SSIB: Swedish Social Insurance Board
